# TiltRec: an ultra-fast and open-source toolkit for cryo-electron tomographic reconstruction

**DOI:** 10.1093/bioinformatics/btaf068

**Published:** 2025-02-14

**Authors:** Yanxin Jiao, Hongjia Li, Yang Xue, Guoliang Yang, Lei Qi, Fa Zhang, Dawei Zang, Renmin Han

**Affiliations:** Qilu Hospital (Qingdao), Cheeloo College of Medicine; Frontiers Science Center for Nonlinear Expectations (Ministry of Education), Research Center for Mathematics and Interdisciplinary Sciences, Shandong University, Qingdao 266237, China; School of Medical Technology, Beijing Institute of Technology, Beijing 100081, China; Qilu Hospital (Qingdao), Cheeloo College of Medicine; Frontiers Science Center for Nonlinear Expectations (Ministry of Education), Research Center for Mathematics and Interdisciplinary Sciences, Shandong University, Qingdao 266237, China; Jinan High-tech Zone Branch Center, Jinan Center for Disease Control and Prevention, Jinan 250101, China; Biomedical Research Center for Structural Analysis, Shandong University, Jinan 250012, China; School of Medical Technology, Beijing Institute of Technology, Beijing 100081, China; High Performance Computer Research Center, Institute of Computing Technology, Chinese Academy of Sciences, Beijing 100190, China; Qilu Hospital (Qingdao), Cheeloo College of Medicine; Frontiers Science Center for Nonlinear Expectations (Ministry of Education), Research Center for Mathematics and Interdisciplinary Sciences, Shandong University, Qingdao 266237, China

## Abstract

**Motivation:**

Cryo-electron tomography (cryo-ET) has revolutionized our ability to observe structures from the subcellular to the atomic level in their native states. Achieving high-resolution reconstruction involves collecting tilt series at different angles and subsequently backprojecting them into 3D space or iteratively reconstructing them to build a 3D volume of the specimen. However, the intricate computational demands of tomographic reconstruction pose significant challenges, requiring extensive calculation times that hinder efficiency, especially with large and complex datasets.

**Results:**

We present TiltRec, an open-source toolkit that leverages the parallel capabilities of Central Processing Units and Graphics Processing Units to enhance tomographic reconstruction. TiltRec implements six classical tomographic reconstruction algorithms, utilizing optimized parallel computation strategies and advanced memory management techniques. Performance evaluations across multiple datasets of varying sizes demonstrate that TiltRec significantly improves efficiency, reducing computational times while maintaining reconstruction resolution.

**Summary:**

TiltRec effectively addresses the computational challenges associated with cryo-ET reconstruction by fully exploiting parallel acceleration. As an open-source tool, TiltRec not only facilitates extensive applications by the research community but also supports further algorithm modifications and extensions, enabling the continued development of novel algorithms.

**Availability and implementation:**

The source code, documentation, and sample data can be downloaded at https://github.com/icthrm/TiltRec.

## 1 Introduction

Cryo-electron tomography (cryo-ET) has emerged as a pivotal technique for the *in situ* examination of cellular and macromolecular architecture, allowing researchers to observe biological structures in their native state (Lučić *et al.* 2005). This technique involves reconstructing a 3D density map from a series of projections collected at different tilt angles, a process that is inherently computationally intensive. A number of tomographic reconstruction methods have been proposed, such as weighted back projection (Radermacher 1992) and iterative reconstruction algorithms (Gilbert 1972, Andersen and Kak 1984). The back-projection technique (Herman 2009a), which is relatively computationally efficient, often results in low reconstruction contrast and anisotropy artifacts. In contrast, iterative methods enhance resolution and reduce noise but require significantly more computational resources. Achieving high resolution with algebraic reconstruction techniques further exacerbates these issues, escalating both computational expense and complexity. Additionally, recent advancements in sample preparation, instrumentation, and data acquisition technologies have led to an exponential increase in data volumes (Zheng *et al.* 2004), presenting substantial challenges in computation times and memory management.

Recently, the adoption of highly parallel architectures, such as Central Processing Units (CPUs) via Message Passing Interface (MPI) and Graphics Processing Units (GPUs) via Compute Unified Device Architecture (CUDA), has significantly transformed computational methods across biological fields (Owens *et al.* 2007). Many current tomographic reconstruction algorithms now incorporate CPU acceleration, such as TOMO3D (Meléndez *et al.* 2015). Similarly, several computational frameworks harness the power of GPUs to provide reconstruction capabilities, including RELION (Zivanov *et al.* 2022), EMAN2 (Rees *et al.* 2013), M (Tegunov *et al.* 2021), and IMOD (Kremer *et al.* 1996). In addition, numerous GPU-optimized reconstruction algorithms have been developed, offering high-performance solutions tailored to tomographic processing (Scherl *et al.* 2007, Schmeisser *et al.* 2009, Noël *et al.* 2010, Xu *et al.* 2010). Despite these advancements, many existing tools continue to rely on outdated hardware architectures, failing to fully leverage the capabilities of modern multi-core CPUs and advanced GPUs. Furthermore, these tools often restrict users’ ability to effectively modify the parameters of 3D volumes, thereby limiting both flexibility and adaptability. The absence of open-source platforms exacerbates these issues, hindering researchers’ ability to customize and optimize algorithms to meet specific experimental needs, thus stifling innovation and tailored problem-solving in the field.

In this work, we develop TiltRec, an integrated and open-source toolkit that fully exploits the parallel capabilities of both CPUs and GPUs for tomographic reconstruction. TiltRec offers parallelized implementations of six classical tomographic reconstruction algorithms, including the Back Projection Technique (BPT) (Herman 2009a), Weighted Back Projection (WBP) (Radermacher 1992), Filtered Back Projection (FBP) (Herman 2009b), Simultaneous Iterative Reconstructive Technique (SIRT) (Gilbert 1972), Simultaneous Algebraic Reconstruction Technique (SART) (Andersen and Kak 1984), and Alternating Direction Method of Multipliers (ADMM). Efficient kernel functions and specific kernel function grid configurations are designed for optimized parallel computation. Additionally, TiltRec offers two memory management strategies: a slice-based memory management strategy that achieves significant acceleration for broad use, and a unified memory management strategy that provides a basic accelerated implementation to facilitate further algorithm development. Additionally, TiltRec supports multiple input and output formats, ensuring broad compatibility with other software platforms. The performance of TiltRec has been validated on three datasets of varying sizes, demonstrating significantly reduced runtimes for all six reconstruction algorithms, even compared to the accelerated versions offered by IMOD and TOMO3D, while still maintaining high reconstruction resolution.

## 2 Materials and methods

**Figure 1.**
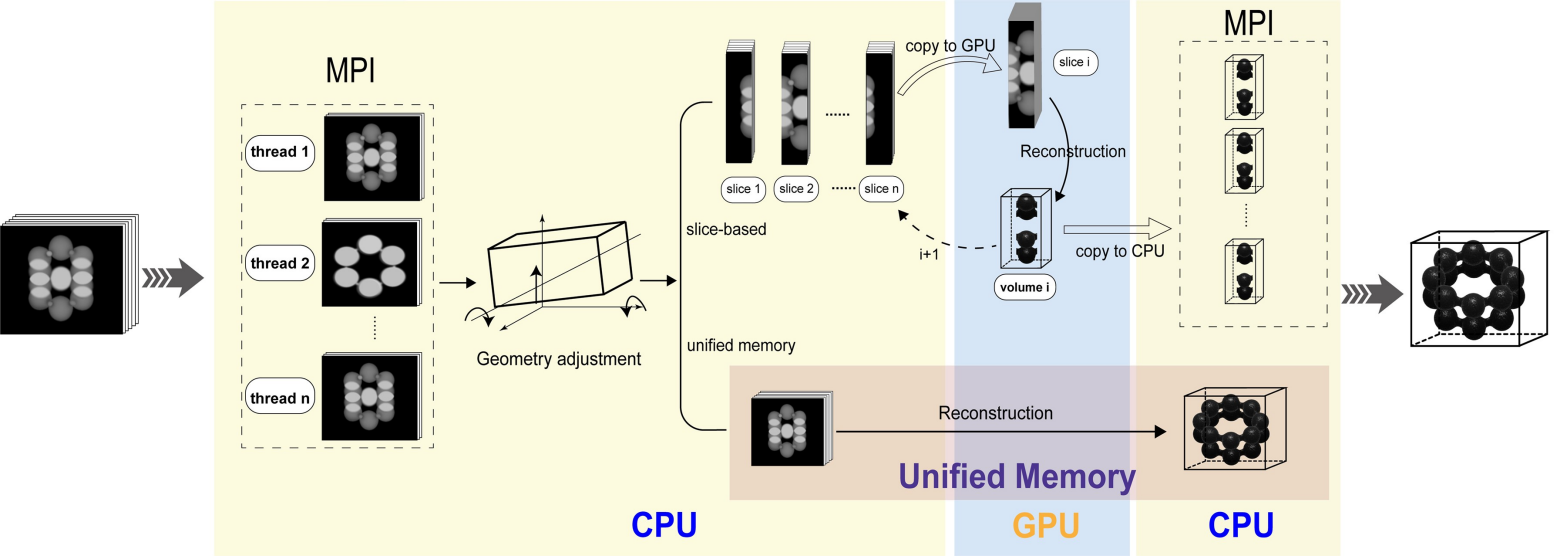
The basic data process workflow in TiltRec. First, distributed file reading is conducted on the CPU through MPI, followed by an adjustment of the sample’s geometry. Subsequently, the computation-intensive reconstruction tasks are performed on the GPU to fully utilize its parallel computing capabilities, with two types of GPU memory management methods. Finally, the reconstructed volumetric data are distributed for output using the CPU.

Figure 1 demonstrates the basic data processing workflow in TiltRec. The input to TiltRec consists of tilt series and their corresponding angles, provided after CTF correction and alignment. TiltRec supports a variety of input formats, including tilt series in ST, HDF, and MRC formats, as well as tilt angle files in TLT and RAWTLT formats. The reconstructed 3D volumes can be output in ST, HDF, or MRC formats. Additionally, TiltRec allows users to customize key parameters such as the number of iterations, relaxation factors, and geometry settings (see Supplementary Section S.2.1.1 for details). TiltRec employs a collaborative programming strategy that integrates MPI (CPU) with CUDA (GPU), optimizing calculation efficiency by leveraging diverse parallel frameworks. As illustrated in Fig. 1, the tomographic reconstruction process for a tilt series includes several key steps: initially, data pre-processing and reading are executed on the CPU through MPI, followed by geometry adjustment. Subsequently, the data are transferred to the GPU for parallelized reconstruction using CUDA. Finally, the reconstructed volume is copied back to the CPU for either combination (parallelized by MPI) or direct output. To support efficient reconstruction, TiltRec provides two memory management strategies: the slice-based memory strategy and the unified memory strategy.

### 2.1 Implementation details of TiltRec toolkit

#### 2.1.1 Geometry model

In TiltRec, once data are loaded into the CPU, a geometric model is utilized to precisely adjust the spatial positioning of the sample within 3D space, ensuring effective and accurate 3D reconstructions. This model, critical for precise “tomogram positioning”, enables users to input specific parameter values, allowing them to customize the sample’s position to meet their unique requirements. TiltRec provides the adjustment for several key parameters, including tilt angle, pitch angle, z shift, and sample thickness (see more details in Supplementary Fig. S1 and Section S1).

#### 2.1.2 Parallel implementation

**MPI-based acceleration:** In TiltRec, the MPI technique is utilized to manage multi-threaded parallel file operations, specifically for reading tilt series and writing combined reconstructed sub-volumes, effectively mitigating I/O bottlenecks. Concurrently, MPI is also used to perform preprocessing tasks, including filtering, radiance computation, and alignment of 3D volumes.

**CUDA-based acceleration:** As shown in Fig. 1, the core computation-intensive tasks of tomographic reconstruction in TiltRec are executed using CUDA to fully leverage the GPU’s parallel processing capabilities. TiltRec optimizes data read efficiency by utilizing specialized GPU memory types, including constant and texture memory. Additionally, customized CUDA kernel functions have been developed based on a voxel splatting strategy, which applies parallel processing strategies at various granularity levels to maximize computational efficiency. To further enhance reconstruction speed, our software dynamically selects the optimal parallel grid configuration during computation, specifically tailored to the unique functionalities and methods of each kernel function (see more design and implementation details in Supplementary Section S4).

#### 2.1.3 Video memory management strategy

To maximize the utilization of CPU and GPU memory, TiltRec provides two memory management strategies: slice-based memory management and unified memory management.

**Slice-based memory management:** In general contexts, the reconstructed 3D volume has no dependencies on directions perpendicular to the projection plane. Therefore, our strategy involves slicing the tilt series along the y-axis within CPU environment. In this way, each slice can thus be treated as independent projection series for subsequent reconstruction (as shown in Supplementary Fig. S2). Upon completion of the slice-grouping process, each slice to be reconstructed is transferred to the GPU environment for parallelized, independent reconstruction operations. Once the reconstruction is complete, the reconstructed subvolume of each slice is transferred back to the CPU for storage and further processing. This process continues until all slices are reconstructed and stacked along the *y*-axis to form the complete 3D volume. It is noteworthy that when the pitch angle is not zero degrees, the slicing direction may no longer be perfectly perpendicular to the projection plane which could result in data voids. To address this issue, we facilitate accurate reconstruction of the 3D volume by filling in these missing areas, as illustrated in Supplementary Fig. S3.

**Unified memory management strategy:** Although the slice-based memory management strategy is efficient, it is not universally applicable to all tomographic reconstruction algorithms, particularly the ADMM. ADMM is an alternating optimization algorithm that relies on the global consistency of the data. When a 3D volume is segmented into multiple slices for parallel processing, the updates for each slice are not fully synchronized with the latest states of other slices, making it challenging to maintain data consistency across the entire volume. This approach can lead to excessive focus on local data within each slice during optimization, thereby neglecting the overall global consistency. Additionally, the inherent characteristics of ADMM’s alternating updates may result in discontinuities at the slice boundaries during the iteration process. Therefore, the ADMM method is not well-suited for a slice-based memory management strategy.

To enhance software versatility and effectively extend video memory, we employ a unified memory strategy that enables parallelism across voxel calculations within the volume. This architecture establishes a single memory address space shared by various processors, including CPUs and GPUs. However, this automated memory management approach can incur additional data transfer overheads, potentially slowing down the reconstruction process compared to slice-based strategies. Moreover, if the CPU memory is also limited, this strategy may become impractical for large datasets. Despite these challenges, this implementation is crucial for datasets that cannot be segmented into slices. Additionally, as a fundamental parallel acceleration strategy, it provides a foundation for further algorithmic modification and extension.

#### 2.1.4 Flexibility and scalability of TiltRec

TiltRec offers four versions of the software: TiltRec-cuda, TiltRec-mpi, TiltRecZ-cuda, and TiltRecZ-mpi.

TiltRec-cuda and TiltRec-mpi are designed for users, utilizing CUDA and MPI for combined acceleration, and solely MPI for acceleration in case GPU is unavailable, respectively. Both versions employ a slice-based memory management strategy. Both TiltRec-cuda and TiltRec-mpi support five tomographic reconstruction algorithms: BPT, WBP, FBP, SIRT, and SART. This slicing-based strategy enables parallelism between voxels within the slice, resulting in significant acceleration. TiltRec-cuda, in particular, is recommended as the preferred choice for users.

TiltRecZ-cuda and TiltRecZ-mpi are designed for developers, utilizing CUDA and MPI for combined acceleration, and solely MPI for acceleration in case GPU is unavailable, respectively. Both versions employ a unified memory management strategy. Both TiltRecZ-cuda and TiltRecZ-mpi support six tomographic reconstruction algorithms: BPT, WBP, FBP, SIRT, SART, and ADMM. These versions do not perform further slicing of the tilt series, instead directly achieving parallelism between voxels within the volume. While the acceleration is not as pronounced as in TiltRec-cuda and TiltRec-mpi, particularly for large datasets, it is essential for implementing algorithms incompatible with slice-based strategies. Additionally, being open-source, these versions offer significant flexibility for developers to further modify and expand the software (see more details in the *Software Manual*).

The implementation details of each reconstruction algorithm are provided in Supplementary Section S1.

## 3 Results

TiltRec offers accelerated implementations for six most commonly used tomographic reconstruction algorithms, including WBP, FBP, SIRT, SART, BPT, and ADMM. For WBP, FBP, and SIRT, both IMOD (Kremer *et al.* 1995) and TOMO3D (TOMO3D 2015) already provide accelerated versions. Consequently, TiltRec’s performance is directly compared with these software tools to evaluate performance enhancements. However, for the other three methods—SART, BPT, and ADMM—the accelerated version of which is not available in existing software, TiltRec’s performance is compared against non-accelerated implementations of these algorithms. TiltRec-cuda and TiltRecZ-cuda are used for comparison. It ensures a comprehensive evaluation of TiltRec’s capabilities across a diverse set of algorithms. To comprehensively evaluate the acceleration performance of our toolkit, we selected three different sizes of datasets for testing. In addition, TiltRec is also compared to commonly used integrated software, such as Relion and EMAN2. For Relion, an additional dataset was used for comparison since Relion is limited to processing only SerialEM data. The results from EMAN2 are shown in Supplementary Table S1. Detailed information about datasets and experimental settings can be found in Supplementary Section S5.

According to the runtimes shown in Table 1, TiltRec-cuda, employing a slice-based memory strategy, consistently delivers the fastest or comparable computation times across nearly all datasets and algorithms. This strategy provides a significant performance boost, achieving acceleration by factors of hundreds when compared to non-accelerated methods. From the “Speedup” column in Table 1, it is evident that TiltRec-cuda offers speedup across all tested methods and datasets. This makes it a viable option for datasets that cannot be efficiently sliced. Additionally, it should be noted that for the large dataset EMPAIR-10045, when the tomographic reconstruction is performed using ADMM, limited by the capacity of CPU, the dataset is first downsampled by a factor of two before reconstruction.

**Table 1.** Runtime comparison for tomographic reconstruction algorithms within different tools (s).

Method	Software	BBb 1024×1024×300	EMPAIR-10453 5760×4092×300	EMPIAR-10045 3838×3710×1081	EMPIAR-10164 7420×7676×300	Speedup^a^
SIRT(30)	TiltRec-cuda	**18.126**	**395.777**	**860.360**	**987.638**	–
	TiltRecZ-cuda	46.563	24 655.766	72 555.030	826 936.478	246.6
	IMOD	55.515	962.735	1848.176	1471.093	2.3
	TOMO3D	237.412	4126.462	6030.4192	2132.804	8.1
FBP	TiltRec-cuda	2.844	282.489	801.271	901.382	–
	TiltRecZ-cuda	**2.843**	730.725	1750.031	15 923.281	5.9
	IMOD	4.178	**254.797**	**714.634**	**852.521**	1.1
	TOMO3D	9.307	452.6952	1018.2589	1002.953	1.8
WBP	TiltRec-cuda	**3.567**	275.172	**820.074**	932.382	–
	TiltRecZ-cuda	3.565	712.965	1505.381	13 029.407	4.8
	IMOD	4.367	**250.408**	837.113	**890.580**	1.0
	TOMO3D	6.018	458.216	1070.4727	932.953	1.4
SART(10)	TiltRec-cuda	**9.816**	**272.983**	**833.109**	**946.145**	–
	TiltRecZ-cuda	16.442	5586.373	17 851.595	241 372.138	74.7
	TiltRec-mpi	15 054.243	351 940.342	291 384.293	94 179.393	818.0
	TiltRecZ-mpi	9828.245	301 141.736	284 105.285	88 103.291	634.6
BPT	TiltRec-cuda	2.984	**248.065**	**735.503**	**822.379**	–
	TiltRecZ-cuda	**2.001**	785.921	1047.275	12 854.158	5.2
	TiltRec-mpi	864.316	27 360.118	26 008.667	59 166.720	126.8
	TiltRecZ-mpi	819.819	26 574.536	26 013.294	59 166.720	122.3
ADMM(10)	TiltRecZ-cuda	**121.370**	**11 067.784**	**789.389** ^b^	**1200.171**	–
	TiltRecZ-mpi	30 385.100	646 805.701	44 118.000^b^	176 041.028^c^	–
	Relion	–	–	–	1726.429^c^	

Bold values mean the best performance.

a The mean ratio of the runtime of other software to that of TiltRec-cuda across each dataset.

b Indicates that result obtained from running on a dataset down-sampled by a factor of two.

c Indicates that result obtained from running on a dataset down-sampled by a factor of four.

Although computational complexity poses a significant challenge in tomographic reconstruction, maintaining high reconstruction resolution is paramount for these algorithms. We also conducted tomographic reconstructions on three distinct datasets using different software platforms. For a focused and reliable comparison, we limited the analysis to three algorithms—SIRT, FBP, and WBP—both IMOD and TOMO3D can reliably execute reconstruction for these algorithms as benchmarking. Supplementary Table S2 presents the *FRC*_0.143_ values of various tomographic reconstruction software. It reveals that the *FRC*_0.143_ values for each tomographic reconstruction algorithm exhibit only minor variations among different implementations. Most notably, TiltRec consistently outperforms other software in terms of *FRC*_0.143_ values on the majority of datasets. This performance underscores TiltRec’s ability not only to enhance calculation efficiency but also to ensure reliable reconstruction resolution(see more details in Supplementary Section S5 and Fig. S5).

## 4 Conclusion

As the volume of data in cryo-ET continues to expand, the demand for accelerated reconstruction processes becomes increasingly critical. Here, we developed TiltRec, a toolkit that leverages a combination of CPU (MPI) and GPU (CUDA) technologies to accelerate computation-intensive tasks in reconstructions. TiltRec integrates six classical reconstruction methods and offers two memory management strategies: slice-based and unified memory management. Additionally, TiltRec provides adjustable geometry parameters to enable more flexible and precise reconstructions. TiltRec achieves significant acceleration compared to both non-accelerated and available accelerated algorithms in existing software.

Our software is available in four versions: TiltRec-cuda, TiltRec-mpi, TiltRecZ-cuda, and TiltRecZ-mpi. TiltRec-cuda and TiltRec-mpi are designed for end-users and employ a slice-based memory reconstruction strategy. TiltRec-cuda utilizes CUDA for enhanced acceleration, while TiltRec-mpi relies solely on MPI, making it suitable when GPU support is unavailable. TiltRec-cuda is particularly noted for its significant computational acceleration, making it an effective tool for reconstruction tasks. On the other hand, TiltRecZ-cuda and TiltRecZ-mpi, which are primarily targeted at developers, adopt a unified memory reconstruction strategy. This approach is less efficient compared to the slice-based strategy but still offers fundamental acceleration. As open-source tools, TiltRecZ-cuda and TiltRecZ-mpi facilitate extensive customization and further development, enhancing their practicality and utility in the field of cryo-ET reconstruction.

## Supplementary Material

btaf068_Supplementary_Data
