# Supplementary material for: TiltRec: an ultra-fast and open-source toolkit for cryo-electron tomographic reconstruction
Source: Bioinformatics. 2025 Feb 14;41(3):btaf068. doi: 10.1093/bioinformatics/btaf068 (PMC11886794; doi:10.1093/bioinformatics/btaf068)
Supplement: btaf068_Supplementary_Data [file btaf068_supplementary_data.zip › TiltRec_sup.pdf]

1. RECONSTRUCTION METHODS

In TiltRec, we have integrated six commonly-used tomographic reconstruction algorithms: BPT, SIRT, SART, FBP, WBP, and ADMM. In the following sections, we will delve into the underlying principles and specific parallelized implementations of each algorithm within our tool. In the following sections, we will delve into the principles of each algorithm as well as their specific implementations within the tool.

A. BPT

The BPT is a fundamental method extensively used to reconstruct a 3D density function from 3D projection tilt series, based on the inverse Radon transform [1]. Practically, BPT involves capturing projections (or Radon transforms) of an object from multiple angles and then redistributing these projections onto a 3D reconstructed image space. Each projection contributes to the final image by distributing its intensity values along the original projection path. By systematically reintegrating these contributions from all sampled angles, BPT effectively reconstructs the original object with high fidelity.

The mathematical expression for BPT is given by the equation:

$$\begin{gathered} f(x,y,z) = \int_0^{2\pi}\int_0^{\pi}\int_0^{\infty} g(\theta,\varphi,s)\delta(*)s^2 \sin\varphi ds d\varphi d\theta, \\ * = x\sin\varphi\cos\theta + y\sin\varphi\sin\theta + z\cos\varphi - s \end{gathered} \tag{S1}$$

where $f(x,y,z)$ denotes the reconstructed density function at point (x,y,z), and $g(\theta,\varphi,s)$ represents the projection data acquired at an angle (θ,φ) and a distance s from the origin,δ is Dirac delta function and the angles (θ,φ) define the direction from the origin to the point in a spherical coordinate system.

In cryo-ET, we adapt Equation S1 by discretizing it to handle a finite set of projection data:

$$x_j = \sum_{i=1}^{m} w_{ij} p_i / \sum_{i=1}^{m} w_{ij}, j = 1, 2, \ldots, N \tag{S2}$$

where N is the number of voxels, m is the number of projection pixels involved in the back-projection. x_j is the intensity value of the jth voxel, p_i represents the intensity value of the ith projection pixel, w_{ij} is the weight that quantifies the contribution of the ith projection pixel to the jth voxel.

This mathematical transform is critical for converting data collected from multiple angles into a coherent image, thereby making BPT indispensable in fields such as medical imaging and materials science. BPT remains to be a popular choice, especially in scenarios where rapid visualization takes precedence over high image fidelity. The pseudo-code of the parallelized BPT algorithm is as follows:

Algorithm S1. Parallel BPT using CUDA

procedure CUBACKPROJECT($p, angles$)
  Initialize array x of size N filled with zeros
  $numerator \leftarrow 0$
  $denominator \leftarrow 0$
  parallel for $j = 1$ to N
    parallel for $i = 1$ to m
      $numerator \leftarrow numerator + w[i][j] \cdot p[i]$
      $denominator \leftarrow denominator + w[i][j]$
    if $denominator \neq 0$
      $x[j] \leftarrow \frac{numerator}{denominator}$
    end for
  end for

However, BPT typically offers only a fuzzy approximation of the actual structure, resulting in reconstructions that are often low in quality and highly sensitive to noise. To overcome these limitations, WBP and FBP employ weighting and convolution techniques on the measured projection images before back-projection. These advanced processing methods significantly mitigate, or even eliminate, the blurring artifacts commonly associated with simple back-projection, thus enabling more precise reconstructions of the underlying three-dimensional structure.

B. WBP

Building upon the foundational principles of the BPT technique, WBP enhances this basic concept by incorporating weighting factors derived from a priori knowledge of the imaging process. Unlike BPT, which does not utilize weighting and simply 'smears' each projection along its direction, resulting in a blurred approximation of the actual structure, WBP strategically applies weights to the projection images before back-projection. This adjustment significantly mitigates the blurring typically observed in the BPT process, leading to clearer and more accurate reconstructions[2].

Based on this idea, WBP can be divided into the following two steps:

- **Weighting:**

$$H(f) = \begin{cases} 0.2 & \text{if } f = 0 \\ f & \text{if } 0 < f \leq \text{weight} \\ \text{weight} \times \exp\left(-\frac{(f-\text{weight})^2}{2\sigma_{Nx}^2}\right) & \text{if } f > \text{weight} \end{cases} \tag{S3}$$

  where $H(\cdot)$ is the function to assign the weight to each projection, σ_{Nx} is the standard deviation for the Gaussian decay portion, and *weight* is a predefined threshold (default as 0.05). It should be noted that the weights for each projection can be calculated using various algorithms, and TiltRec provides one possible implementation.

- **Back-projection:** Similar to the BPT process.

Through its adaptive integration of these weighted contributions, WBP not only mitigates artifacts and enhances resolution but also facilitates the reconstruction of intricate details within the specimen, unraveling the structural complexities inherent to cryo-ET investigations. The pseudo-code of the parallelized WBP algorithm is as follows:

Algorithm S2. Parallel WBP using CUDA

procedure CUWBP(p, $angles$)
  Initialize array x of size N filled with zeros
  Calculated the weighted projections, $\tilde{p}$, with weighting function $H(\cdot)$
  $numerator \leftarrow 0$
  $denominator \leftarrow 0$
  parallel for $j = 1$ to N
    parallel for $i = 1$ to m
      $numerator \leftarrow numerator + w[i][j] \cdot \tilde{p}[i]$
      $denominator \leftarrow denominator + w[i][j]$
    if $denominator \neq 0$
      $x[j] \leftarrow \frac{numerator}{denominator}$
    end for
  end for

C. FBP

Similar to WBP algorithm, the FBP technique relies on a Fourier-based filtering approach applied to the projections, aiming to eliminate artifacts and improve structural details in the resulting volume reconstructions. In our software, two filters are provided, RamLak filter and SheppLogan filter. The RamLak filter is particularly effective in suppressing high-frequency noise while preserving low-frequency information in the frequency domain, thereby enhancing the clarity of reconstructed images. Conversely, the Shepp-Logan filter is designed to minimize artifact occurrences, leading to more precise and accurate reconstructions. By incorporating these filters into the FBP reconstruction process, our software empowers users to tailor the reconstruction pipeline to their specific imaging requirements, ultimately facilitating the production of high-quality cryo-ET reconstructions with improved structural fidelity and reduced artifacts.

Based on this idea, WBP can be divided into the following two steps:

- **Filtering:**

$$H_{RL}(\omega) = \begin{cases} 2\omega/\omega_c, & 0 \leq \omega \leq \omega_c \\ 0, & \textit{otherwise} \end{cases} \tag{S4}$$

$$H_{SL}(\omega) = \begin{cases} \frac{2}{\pi}\left[\frac{\sin\left(\frac{\pi}{2}\frac{\omega}{\omega c}\right)}{\frac{\pi}{2}\frac{\omega}{\omega c}}\right]^2, & |\omega| \leq \omega_c \\ 0, & otherwise \end{cases} \tag{S5}$$

where K is the number of the projections in tilt series, F is the Fourier transform, F^{-1} is the inverse Fourier transform, $H(\cdot)$ is the filter function where $H_{RL}(\cdot)$ indicates the RamLak filter and $H_{SL}(\cdot)$ indicates the SheppLogan filter, ω represents frequency and ω_c is the cutoff frequency.

- **Back-projection:** Similar to the BPT process.

The pseudo-code of the FBP algorithm is as follows:

Algorithm S3. Parallel FBP using CUDA

procedure CUFBP($p, angles$)
    Initialize array x of size N filled with zeros
    Initialize array H with RamLak or Shepp-Logan filter values
    Convolve projection with filter H, obtain $\tilde{p}$
    $numerator \leftarrow 0$
    $denominator \leftarrow 0$
    parallel for $j = 1$ to N
        parallel for $i = 1$ to m
            $numerator \leftarrow numerator + w[i][j] \cdot \tilde{p}[i]$
            $denominator \leftarrow denominator + w[i][j]$
        if $denominator \neq 0$
            $x[j] \leftarrow \frac{numerator}{denominator}$
        end for
    end for

D. SIRT

Unlike conventional back projection-based approaches, SIRT adopts an iterative strategy, refining the reconstructed volume through successive iterations. The SIRT algorithm starts with an initial guess for the reconstruction and progressively updates it to minimize the error between the calculated projections of the current reconstruction and the actual observed projections. The SIRT algorithm can be represented by the following equation:

$$p_i^* = \sum_{j=1}^{n} w_{ij}^{(k)} x_j^{(k)} \quad 1 \leq i \leq M \tag{S6}$$

$$x_j^{(k+1)} = x_j^{(k)} + \frac{\lambda}{\sum_{i=1}^{m} w_{ij}^{(k+1)}} \sum_{i=1}^{m} w_{ij}^{(k+1)} (p_i - \frac{p_i^*}{\sum_{k=1}^{n} w_{ik}^{(k)}}) \quad 1 \leq j \leq N \tag{S7}$$

where p_i^* represents the intensity value of the ith projection pixel of at iteration k, x_j^k is the value of jth voxel at iteration k, $x_j^{(k+1)}$ is the updated value of jth voxel at iteration $k+1$, $w_{ij}^{(k)}$ and $w_{ij}^{(k+1)}$ indicate the weights representing the contribution of jth voxel to the ith projection pixel at iteration k and $k+1$, respectively. p_i denotes the measured intensity value of the ith projection pixel, λ is is a regularization parameter controlling the extent of updates at each iteration. n is the number of voxels used for projection and is a subset of all voxels, N is the number of voxels and M is the number of projection pixels

The simultaneous nature of SIRT enables the algorithm to globally optimize the reconstruction, making it well-suited for handling noisy or incomplete data while preserving fine structural details. The pseudo-code of the SIRT algorithm is as follows:

E. SART

SART is another iterative reconstruction method, but it deviates from SIRT in its approach to updating the reconstructed volume. While SIRT takes a global approach by considering all rays from various angles, SART uses an ordered subset strategy.

Algorithm S4. Parallel SIRT using CUDA

procedure CUSIRT($p, angles, \lambda, N_{\text{iter}}$)
  Initialize voxel vector $x^{(0)}$ with zeros
  for $k = 1$ to N_{iter} **do**
    parallel for $i = 1$ to M
      $p_i^* \leftarrow \sum_{j=1}^{n} w_{ij} x_j$
    end for ▷ Forward Projection
    parallel for $i = 1$ to M
      $r_i \leftarrow p_i - \frac{p_i^*}{\sum_{h=1}^{n} w_{ih}}$ ▷ Calculate Projection Difference
    end for
    parallel for $j = 1$ to N
      $y_j \leftarrow \sum_{i=1}^{m} w_{ij} r_i$ ▷ Back Projection
    end for
    parallel for $j = 1$ to N
      $x_j^{(k+1)} \leftarrow x_j^{(k)} + \lambda \frac{y_j}{\sum_{i=1}^{m} w_{ij}}$ ▷ Update
    end for

In SART, the correction for each voxel is determined by considering all rays that pass through that voxel but only from the same projection angle, that is a subset of the total rays. The underlying mathematical representation and update formula remain similar to those of SIRT, shown as follows:

$$p_i^* = \sum_{j \in s} w_{ij}^{(k)} x_j^{(k)} \quad 1 \le i \le M \tag{S8}$$

$$x_j^{(k+1)} = x_j^{(k)} + \lambda \sum_{s \subset M} \left(\frac{1}{\sum_{i \in s} w_{ij}^{(k+1)}} \sum_{i \in s} w_{ij}^{(k+1)} (p_i - \frac{p_i^*}{\sum_{k=1}^{n} w_{ik}^{(k)}}) \right) \quad 1 \le j \le N \tag{S9}$$

where s is a subset of all projections M. The focus of SART technique is on a localized region to update, making the process more sensitive to the specific contributions from that region.The pseudo-code of the SART algorithm is as follows:

Algorithm S5. Parallel SART using CUDA

procedure CUSART($p, angles, \lambda, N_{\text{iter}}$)
  Initialize voxel vector $x^{(0)}$ with zeros
  for $k = 1$ to N_{iter} **do**
    for each subset s **do**
      parallel for i in s
        $p_i^* \leftarrow \sum_{j=1}^{n} w_{ij} x_j$ ▷ Forward Projection
      end for
      parallel for i in s
        $r_i \leftarrow p_i - p_i^*$ ▷ Calculate Projection Difference
      end for
      parallel for $j = 1$ to N
        $y_j \leftarrow \sum_{i \text{ in } s} w_{ij} r_i$ ▷ Back Projection
      end for
      parallel for $j = 1$ to N
        $x_j^{(k+1)} \leftarrow x_j^{(k)} + \lambda \frac{y_j}{\sum_{i \text{ in } s} w_{ij}}$ ▷ Update
      end for
  return $x^{(N_{\text{iter}})}$

The benefit of SART lies in its efficiency and accuracy. It can achieve good reconstruction quality with fewer iterations. However, this comes at the expense of computational resources, as SART generally requires more processing power to handle the localized updates.

F. ADMM

ADMM, is an iterative algorithm used to solve optimization problems. The algorithm decomposes complex optimization problems into a series of simpler sub-problems, which are then solved individually. In tomographic reconstruction, variable splitting can separate the data fidelity terms and regularization terms in the reconstruction problem. Specifically, the tomographic reconstruction task can be formulated as an optimization problem in the following form:

$$\hat{\mathbf{x}} = \arg\min \left\{ \frac{1}{2} \|\mathbf{W}\mathbf{x} - \mathbf{p}\|_2^2 + \lambda \mathscr{R}(\mathbf{L}\mathbf{x}) \right\}, \tag{S10}$$

where $\hat{\mathbf{x}} \in \mathbb{R}^N$ is the recovered volume estimate, $\mathscr{R} : \mathbb{R}^Q \to \mathbb{R}$ is a regularization functional, $\mathbf{L} \in \mathbb{R}^{Q \times N}$ is a regularization operator, and $\lambda > 0$ is the regularization parameter. A popular prior is the edge-preserving total-variation (TV) regularization $\mathscr{R} = \| \cdot \|_{1,2}, \mathbf{L} = \nabla$.

By introducing an auxiliary variable $\mathbf{u} = \mathbf{L}\mathbf{x}, \mathbf{u} \in \mathbb{R}^Q$, we can rewrite the equation as a constrained optimization problem:

$$\begin{aligned} \hat{\mathbf{x}} &= \arg\min_{\mathbf{x} \in \mathbb{R}^N} \frac{1}{2} \|\mathbf{W}\mathbf{x} - \mathbf{p}\|_2^2 + \lambda \mathscr{R}(\mathbf{u}), \\ \text{s.t.} &\quad \mathbf{u} = \mathbf{L}\mathbf{x}. \end{aligned}$$

Its augmented Lagrangian function is given by

$$\mathscr{L}(\mathbf{x}, \mathbf{u}, \boldsymbol{\alpha}) = \frac{1}{2} \|\mathbf{p} - \mathbf{W}\mathbf{x}\|_2^2 + \lambda \mathscr{R}(\mathbf{u}) + \boldsymbol{\alpha}^T (\mathbf{L}\mathbf{x} - \mathbf{u}) + \frac{\rho}{2} \|\mathbf{L}\mathbf{x} - \mathbf{u}\|_2^2, \tag{S11}$$

where $\boldsymbol{\alpha} \in \mathbb{R}^Q$ is the vector of Lagrange multipliers, and $\rho > 0$ is a penalty parameter that influences the convergence speed.

The complete reconstruction method is summarized in Algorithm S6 below.

Algorithm S6. Parallel ADMM using CUDA

```
procedure CuADMM(p, angles, λ, N_iter, N_cg, threshold)
    Initialize u^0 = Lc^0, α^0 = u^0
    for k = 1 to N_iter do
        Parallelize the computation of x^(k+1) ← solve (5) using Conjugate Gradient
        Parallelize the computation of u^(k+1) = softthreshold_R (Lx^(k+1) − α^(k)/ρ ; λ/ρ)
        Parallelize the computation of α^(k+1) = α^(k) + ρ (Lx^(k+1) − u^(k+1))
```

2. GEOMETRY MODEL

Accurate positioning of samples in three-dimensional space is crucial, especially during the process of precise 3D reconstruction. In TiltRec, geometric parameters play a critical role in the reconstruction process, as they represent displacements in various directions, enabling users to apply offsets to tilt angles and perform rotations of the tomogram around the tilt axis. This adjustment can make slice reconstructions appear more horizontal, which is particularly useful for specific types of reconstructions. This model is essential for precise 'tomogram positioning', as it allows users to input specific parameter values to customize the sample's position according to their unique requirements.

Furthermore, it helps to focus the reconstruction on regions of interest, thus reducing processing time. However, caution is necessary when using this parameter for subtomogram averaging. The offset may cause the missing wedge region to rotate around the tilt axis, potentially affecting the final averaged results. Therefore, when inputting missing wedge information into subtomogram averaging packages, users must be particularly careful to avoid introducing errors or biases.

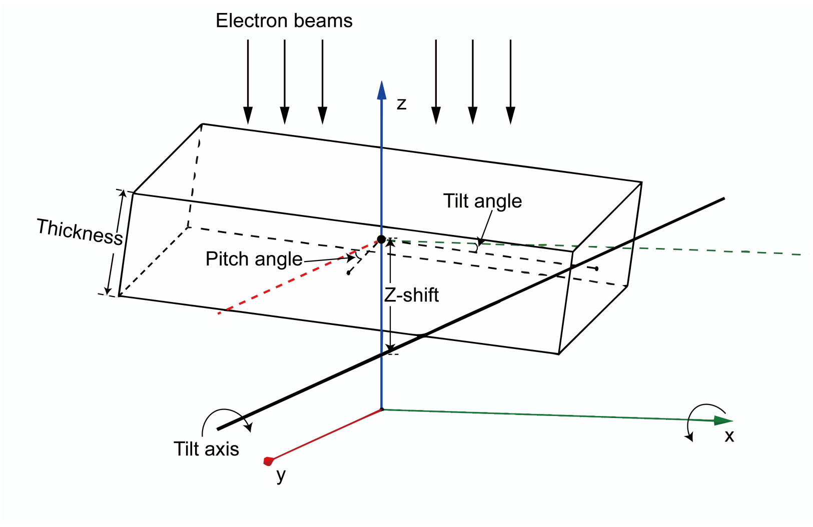

Fig. S1. Schematic diagram of the geometry model. The geometry parameters mainly includes tilt angle, pitch angle, z shift and thickness.

The geometry model used in our tool is detailed in Figure S1 and includes the following four key parameters:

1. **Tilt Angle:** Defines the rotation of the specimen around the tilt axis.
2. **Pitch Angle:** Represents the rotation angle of the specimen around the x-axis.
3. **Z-Shift:** Specifies the distance from the tilt axis to the specimen's center in the z-direction.
4. **Thickness:** Indicates the specimen's thickness.

By manipulating these parameters, users can achieve precise control over the spatial orientation and positioning of the specimen, thereby improving the accuracy of the resulting 3D reconstructions.

3. SLICE-BASED MEMORY MANAGEMENT

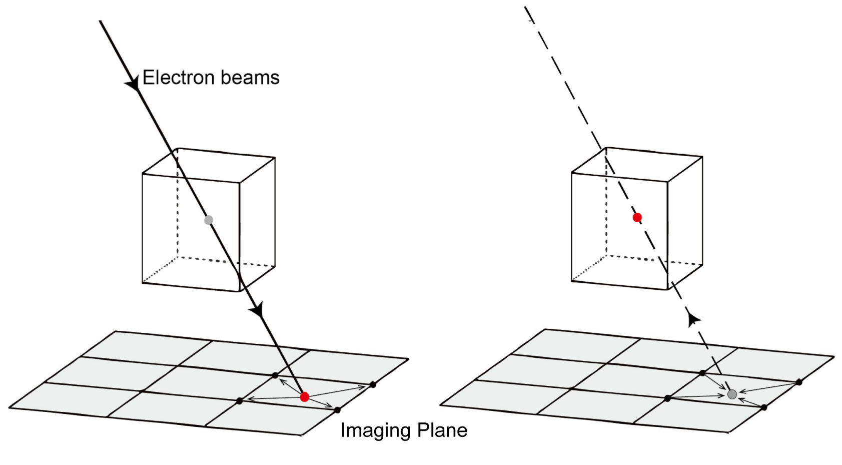

Fig. S2. Slice Scheme of a Three-Dimensional Volume on the YOZ Plane. The white area represents the final output, while the gray area indicates regions anticipated to require supplementation to ensure accuracy in reconstruction.

Since the projection images don't have any dependencies along directions perpendicular to the projection plane, such as the x-axis and y-axis, we have adopted a strategy of slice-grouping along the y-axis. Specifically, this approach initially segments a series of inclined images into slices along the y-axis within a CPU environment. Each slice can thus be treated as an independent two-dimensional projection for subsequent reconstruction. As depicted in S2, such a volume in the XOZ plane is decomposed into n distinct slices.

It is noteworthy that when the pitch angle is not equal to zero degrees, the slicing direction may no longer be perfectly perpendicular to the projection plane. In such scenarios, slicing along the y-axis could result in data voids. To address this issue, we facilitate the accurate reconstruction of the three-dimensional volume by filling in these missing areas, as illustrated in Figure S3. Therefore, each slice comprises both the reconstruction region (the white portions in Figure S2 and Figure S3) and the supplemented region (the gray portions in Figure S2 and Figure S3).

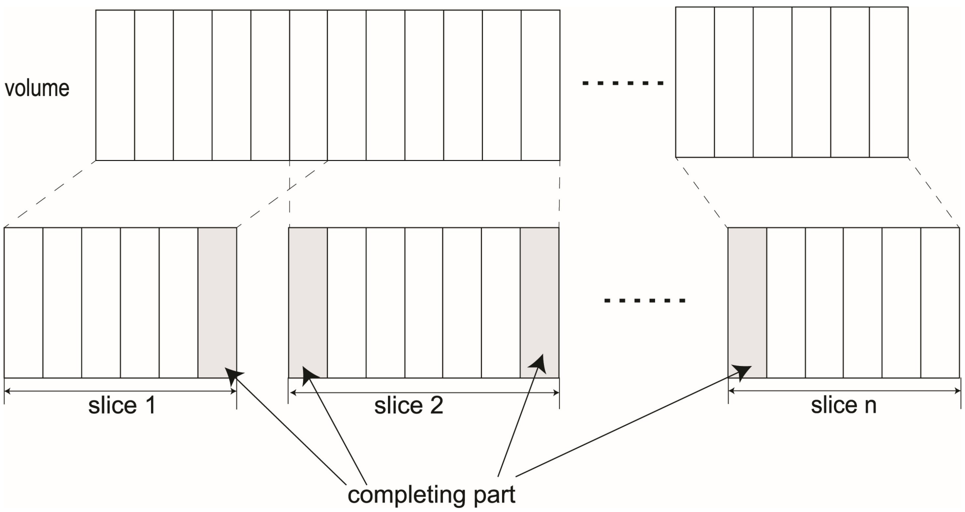

Fig. S3. Schematic of a Sliced Tomography in 3D Space. The white area represents the final output, while the gray area indicates regions anticipated to require supplementation to ensure accuracy in reconstruction.

Upon completion of the slice-grouping process, the data block to be reconstructed is transferred to a GPU environment to perform parallelized, independent reconstruction operations. During each iteration cycle, an individual slice is reconstructed on the GPU. Once the reconstruction is complete, the data for that slice is transferred back to the CPU for storage and further processing. The algorithm then proceeds to reconstruct the next slice, continuing this process until all slices have been fully reconstructed. Ultimately, all individually reconstructed slices are stacked along the y-axis to form a complete three-dimensional volume.

4. CUDA-BASED ACCELERATION

TiltRec leverages CUDA for core computation-intensive tasks in tomographic reconstruction, optimizing data read efficiency through specialized GPU memory types and employing customized CUDA kernel functions based on a voxel splatting strategy. The software dynamically selects optimal parallel grid configurations to enhance computational efficiency and reconstruction speed.

Kernel function implementation. In the reconstruction process, the projection and back-projection steps are typically the most time-consuming. Our program employs a voxel splatting strategy, as illustrated in Figure S4. In this approach, each voxel is "distributed" onto the detector, updating the pixel values at four adjacent points, which ultimately generates a projection image. These same four points in the projection data are then used to update the voxel in the reconstructed volume. Both projection and back-projection utilize transposed operations to enhance the accuracy of the results. This strategy simplifies calculations and accelerates the process compared to traditional ray-casting methods [3].

Kernel function grid configuration. Choosing the appropriate grid and block sizes is critical not

only for ensuring correct computational results but also for the efficient utilization of Streaming Multiprocessors (SMs) on GPUs. Optimal grid and block configurations enhance parallelism and increase resource occupancy, leading to better overall performance. Below are the grid configurations for the main kernel functions used in our application.

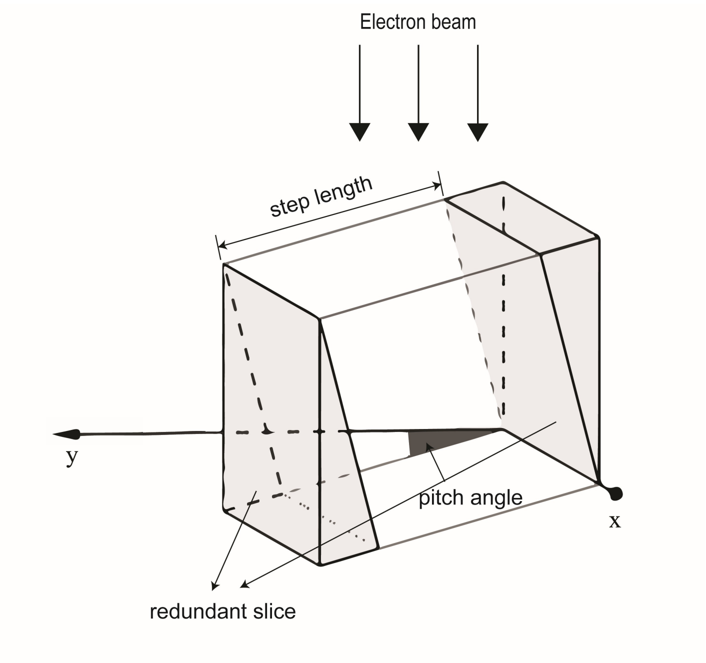

Fig. S4. Schematic diagram of the voxel splatting strategy. Schematic diagram of the voxel splatting strategy. The left figure illustrates the projection process, where voxels are "splatted" onto the imaging plane, updating the pixel values at four adjacent points to create the projection image. The right figure depicts the back-projection process, which is the transpose of the projection process, reversing the operation to reconstruct the volume from the projections.

Forward Projection: Use a two-dimensional(2D) grid to generate projection images. The size of the grid is calculated from two parameters: the first dimension is determined by dividing the number of voxels on the side of the volume by the number of threads per thread block, and the other is the number of projections (if the value is 1, it is a one-dimensional grid). To enhance computational efficiency, each thread within the grid is responsible for computing the projections for a specific section of the volume. These threads iterate along the y-direction to compute projection values, thereby reducing the time required to calculate projection coordinates.
Calculation of Difference: A 2D grid is employed to compute the differences between the calculated and actual projections. The grid dimensions are determined by two parameters: the number of pixels in the projected image for one dimension, and the number of projections for the other dimension. Each thread is responsible for calculating the difference between a pixel point and its corresponding actual projection.
Back projection: A 3D grid is utilized for back-projection tasks. The dimensions of the grid are calculated based on three parameters: the width, thickness, and length of the volume. This ensures that the grid accurately represents the entire volume to be reconstructed. To optimize processing and avoid computational conflicts, each thread within this grid is assigned a specific voxel. It is then responsible for calculating the back-projection values for this voxel across all projection angles. This approach enhances efficiency by distributing the computational load evenly across multiple threads.
Update the volume: A one-dimensional grid is used to update the 3D volume, with the number of threads in the grid equal to the total number of voxels in the volume. Each thread is responsible for updating a single voxel.

5. EXPERIMENTS

In the supplemental data, the performance of the TiltRec software is validated through two key aspects:

First, we evaluate the precision of reconstructions achieved by these algorithms. This step is crucial to demonstrate that TiltRec maintains high quality in reconstruction, comparable to or exceeding that of existing solutions. Secondly, we explore how different settings of geometry parameters influence the reconstruction results. This analysis will show TiltRec's flexibility and adaptability in handling different imaging conditions and requirements. Through these evaluations, we aim to comprehensively demonstrate TiltRec's robustness and effectiveness in improving both the speed and quality of tomographic reconstructions.

All experiments were conducted on a computer equipped with an Intel Xeon Gold 6258R processor featuring 112 threads across all cores and sockets, an NVIDIA RTX 3090 graphics card with 24 GB of video memory and CUDA version 12.4. For the software configurations, IMOD was set to utilize its default GPU acceleration, optimizing for the best performance possible on the available hardware. Conversely, TOMO3D was operated using its default setting of 20 threads, leveraging multi-threading to enhance processing speed. For the TiltRec, four threads were utilized during the slice-based reconstruction, while a single thread was employed for testing unified memory-based reconstruction.

A. Dataset information

To comprehensively validate the efficiency of TiltRec, three public datasets of varying sizes were selected: BBb[4], EMPAIR-10453[5], and EMPAIR-10045[6].

Dataset BBb: This dataset is a tilt series of plastic embedded cell sections around a centriole region, captured using a FEI TF30 microscope operating at 300 kV with a Gatan Camera. It was sourced from the IMOD tutorial, available at IMOD tutorial data https://bio3d.colorado.edu/imod/betaDoc/binspec.html.

The tilt angles range from $+65.0^\circ$ to -65.0° at 2° intervals, comprising 64 images per tilt series. Each image measures 1024x1024 pixels, with a pixel size of 1.01 nm. The reconstructed 3D volume is 1024x1024x300 pixels, occupying approximately 1.2 GB of memory.

Dataset EMPAIR-10453: This dataset consists of 41 tilt series in MRC image format. The tilt angles range from -60° to 60° at 3° intervals. Each image measures 5760x4092 pixels and uses UNSIGNED 16 BIT INTEGER for pixel type. The pixel spacing is 1.329 A in both dimensions.

The reconstructed dataset occupies approximately 27 GB of memory with a reconstruction thickness of 300 pixels. This dataset can be downloaded at https://www.ebi.ac.uk/empiar/EMPIAR-10453/.

Dataset EMPAIR-10045: This dataset is a tilt series of purified brewing yeast 80S ribosomes. The tilt angles range from -57.9° to 29.4° at 3.0° intervals, comprising 31 images per tilt series. Each image measures 3838x3710 pixels.

The reconstructed 3D volume occupy approximately 60 GB of memory with a reconstruction thickness of 1081 pixels. This dataset serves as a rigorous test for CUDA-accelerated reconstruction software, particularly given its high memory requirements that exceed the capacity of most GPUs. This dataset can be downloaded at https://www.ebi.ac.uk/empiar/EMPIAR-10045/

Dataset EMPAIR-10164: This subset of the EMPAIR-10064 dataset contains five selected tilt series, including TS_01, TS_03, TS_43, TS_45, and TS_54. These tilt series correspond to immature HIV-1 dMACANC virus-like particles (VLPs) assembled in the presence of BVM and were acquired using an EMBL Titan Krios electron microscope with SerialEM software.

In this study, we specifically selected `TS_01`, which consists of 41 images with dimensions of (7420×7676). The tilt angles for this series range from 60.0006° to -59.9986°. After reconstruction, the resulting dataset size is `63.7 GB`, and it can be accessed via the following link: https://www.ebi.ac.uk/empiar/EMPIAR-10164/.

B. Comparison of Reconstruction Time Using EMAN2

Due to the specific reconstruction methods employed by EMAN2, and the limited output sizes it provides (e.g., 1k or 2k), we conducted an additional experiment to compare the total reconstruction time across the four datasets used. The results of this experiment are presented in Table S1. From the data in the table, it can be seen that our software has a significant advantage in reconstruction speed.

Table S1. Comparison of Reconstruction Time Using EMAN2(seconds).

Software	BBb 1000 × 1000 × 300	EMPAIR-10453 2000 × 2000 × 300	EMPIAR-10045 2000 × 2000 × 1081	EMPIAR-10164 2000 × 2000 × 300
Eman2	150.3	405.0	413.4	528.3

C. Reconstruction result

Although computational complexity poses a significant challenge in tomographic reconstruction, maintaining high reconstruction resolution is paramount for these algorithms. To verify that our software maintains the essential reconstruction resolution, we conducted tomographic reconstructions on three distinct datasets using different software platforms. For a focused and reliable comparison, we limited the analysis to three algorithms—SIRT, FBP, and WBP— both IMOD and TOMO3D can reliably execute reconstruction for these algorithms as benchmarking.

In addition to the classical reconstruction approaches already mentioned, various other software frameworks—such as RELION [7] and EMAN2 [8]—offer tomographic reconstruction functionalities as integrated modules rather than standalone algorithms. To achieve a more comprehensive assessment, we have included these packages in our performance comparisons as well. However, due to RELION's current restriction of only handling SerialEM data, our tests involving RELION were limited to the fourth dataset.

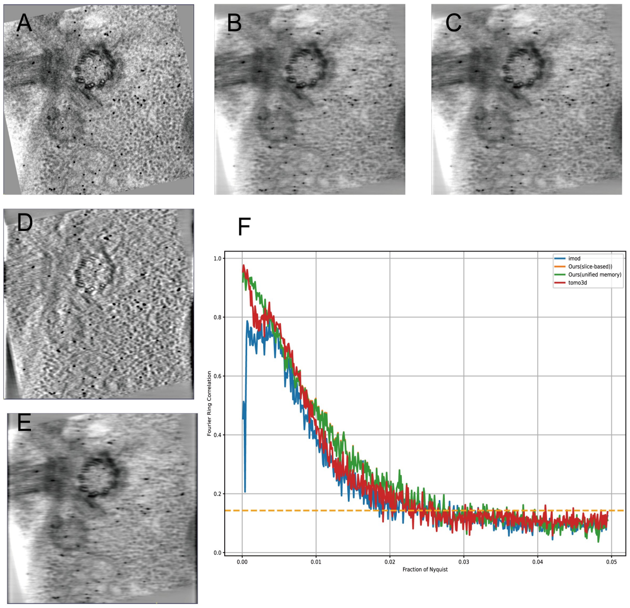

Fig. S5. Comparative analysis of reconstruction results using Fourier Ring Correlation (FRC) curves for SIRT technique. (A) Ground Truth. (B) Reprojection generated by TiltRec-cuda.(C) Reprojection generated by TiltRecZ-cuda. (D) Reprojection generated by IMOD. (E) Reprojection generated by TOMO3D. (F) FRC Curve Comparison: depicting the FRC curves comparing the reprojection generated by TiltRec-cuda, TiltRecZ-cuda, IMOD, and TOMO3D with the Ground Truth.

To minimize subjective biases from visual interpretation, we adopt an 'omit reconstruction' strategy for comparison [9]. This method involved initially removing the projection image at the lowest angle (0 degree) from each dataset, using this image as a 'ground truth' benchmark. We then reconstructed the volume from the remaining data. These reconstructed volumes were subsequently re-projected at the omitted angle to create new projection images. The effectiveness of the different reconstruction methods was quantitatively assessed by comparing the Fourier Ring Correlation (FRC) curves of these re-projected images against the original omitted image.

Table S2. Fraction values corresponding to $FRC_{0.143}$ for different data software.

Method	Software	Fraction values		
		BBb	EMPAIR-10453	EMPIAR-10045
SIRT(10)	TiltRec-cuda	**0.023**	**0.011**	**0.019**
	TiltRecZ-cuda	**0.023**	**0.011**	**0.019**
	IMOD	0.018	**0.011**	**0.019**
	TOMO3D	0.019	**0.011**	**0.019**
FBP	TiltRec-cuda	**0.106**	**0.008**	**0.005**
	TiltRecZ-cuda	**0.106**	**0.008**	**0.005**
	IMOD	0.063	0.007	0.004
	TOMO3D	0.092	0.007	0.003
WBP	TiltRec-cuda	0.070	**0.007**	**0.003**
	TiltRecZ-cuda	0.070	**0.007**	**0.003**
	IMOD	0.056	**0.007**	**0.003**
	TOMO3D	**0.080**	**0.007**	**0.003**

The higher frequency indicates a closer approximation to the ground truth, suggesting better reconstruction quality (the best results are highlighted in bold).

Figure S5 displays the 'ground truth' and the reconstruction results of SIRT algorithm implemented in various tomographic softwares for the dataset BBb, as well as their corresponding FRC curves. Despite some variations in the visualization of the results, the FRC curves from the three software programs exhibit notable similarities, indicating a consistent level of performance across different reconstruction methods. To quantitatively assess the resolution, the fraction of the Nyquist limit at which the FRC reaches 0.143 (denoted as $FRC_{0.143}$) serves as a metric for reconstruction accuracy. Although this does not provide the exact physical resolution, it is a valuable metric for comparing the relative reconstruction capabilities of different algorithms, where the larger value indicates better performance.

Table S2 presents the $FRC_{0.143}$ values of various tomographic reconstruction software across three distinct datasets. The table reveals that the $FRC_{0.143}$ values for each tomographic reconstruction algorithm exhibit only minor variations among different implementations. Most notably, TiltRec (both TiltRec-cuda and TiltRecZ-cuda) consistently outperforms other software in terms of $FRC_{0.143}$ values on the majority of datasets. This performance underscores TiltRec's ability not only to enhance calculation efficiency but also to ensure reliable reconstruction resolution. However, it can be found that within the same dataset, different reconstruction algorithms yield varying $FRC_{0.143}$ values with difference cannot be ignored. It should be noted that these discrepancies stem solely from the intrinsic characteristics of the algorithms themselves, independent of any effects related to parallelization implementation. Figure S6 illustrates a cross-section of the volume reconstructed using six distinct reconstruction algorithms provided by TiltRec-cuda(BPT, WBP, FBP, SIRT and SART) and TiltRec-cuda (ADMM). It can be seen that significant differences are also evident in the visual reconstruction results produced by different algorithms, aligning with the variations observed in the FRC $FRC_{0.143}$ values.

D. Reconstruction with different geometry parameters

To assess the impact of geometric parameters on reconstruction outcomes, we performed reconstructions of the dataset BBb under various geometric configurations using the SIRT algorithm implemented in TiltRec-cuda. The details of four different sets of geometric parameters are presented in Table S3. For each configuration, the thickness parameter was held constant at 300, while the tilt angle, pitch angle, and z-shift were varied to allow a comprehensive comparative

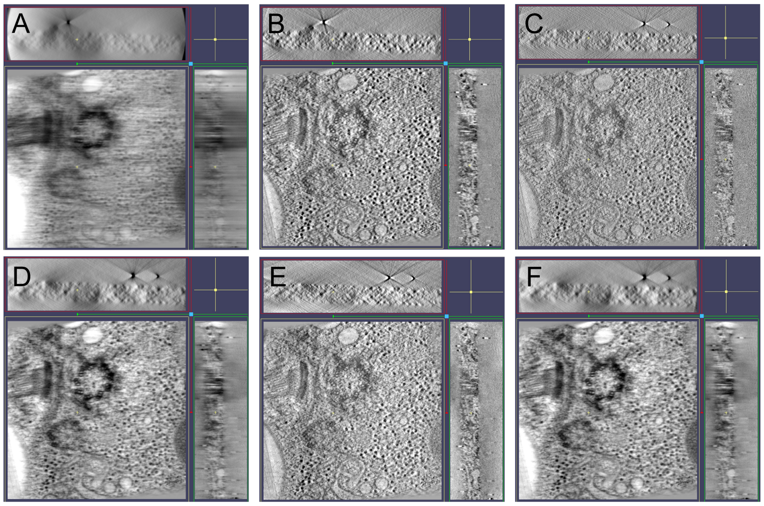

Fig. S6. Comparative analysis of reconstruction results using different tomographic reconstruction algorithms implemented by TiltRec-cuda. (A) BPT. (B) WBP. (C) FBP. (D) SIRT. (E) SART. (F) ADMM (provided by TiltRecZ-cuda).

analysis.

Table S3. Four different geometric parameters settings.

	Tilt angle	Pitch angle	Z-shift	Thickness
A	0	0	0	300
B	10	0	0	300
C	0	10	0	300
D	0	10	10	300

The reconstruction results are displayed in Figure S7. It can be observed that under various geometry parameter settings, the reconstructed structures exhibit different levels of distortion. This variation underscores the critical importance of accurately selecting geometry settings to achieve optimal reconstruction outcomes.

REFERENCES

1. Peter Toft. The radon transform. *Theory and Implementation (Ph. D. Dissertation)(Copenhagen: Technical University of Denmark)*, 1996.
2. Joachim Frank. *Electron tomography: methods for three-dimensional visualization of structures in the cell*. Springer Science & Business Media, 2008.
3. Klaus Mueller and Roni Yagel. Fast perspective volume rendering with splatting by utilizing a ray-driven approach. In *Proceedings of Seventh Annual IEEE Visualization'96*, pages 65–72. IEEE, 1996.
4. James R Kremer, David N Mastronarde, and J Richard McIntosh. Computer visualization of three-dimensional image data using IMOD. *Journal of structural biology*, 116(1):71–76, 1996. Publisher: Elsevier.
5. Beata Turoňová, Mateusz Sikora, Christoph Schürmann, Wim J. H. Hagen, Sonja Welsch, Florian E. C. Blanc, Sören von Bülow, Michael Gecht, Katrin Bagola, Cindy Hörner, Ger van Zandbergen, Jonathan Landry, Nayara Trevisan Doimo de Azevedo, Shyamal Mosalaganti, Andre Schwarz, Roberto Covino, Michael D. Mühlebach, Gerhard Hummer, Jacomine Krijnse

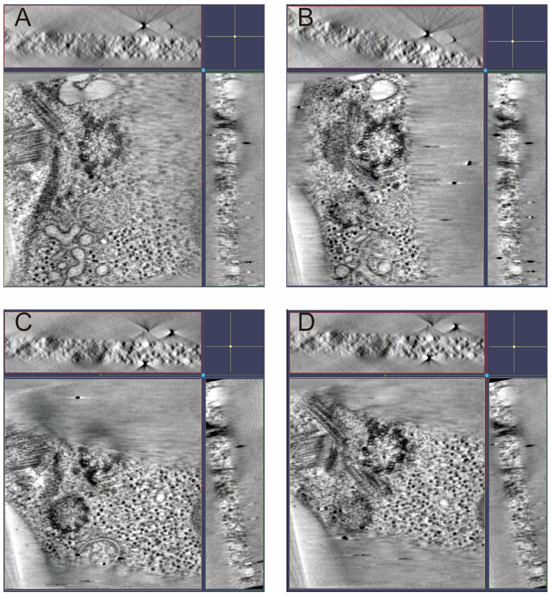

Fig. S7. Visualization of reconstruction results for different geometric parameters. The SIRT algorithm implemented in TiltRec-cuda is employed for reconstruction.

Locker, and Martin Beck. In situ structural analysis of sars-cov-2 spike reveals flexibility mediated by three hinges. *Science*, 370(6513):203–208, 2020.

6. Tanmay AM Bharat and Sjors HW Scheres. Resolving macromolecular structures from electron cryo-tomography data using subtomogram averaging in relion. *Nature protocols*, 11(11):2054–2065, 2016.
7. Jasenko Zivanov, Joaquín Otón, Zunlong Ke, Andriko von Kügelgen, Euan Pyle, Kun Qu, Dustin Morado, Daniel Castanño Díez, Giulia Zanetti, Tanmay AM Bharat, et al. A bayesian approach to single-particle electron cryo-tomography in relion-4.0. *Elife*, 11:e83724, 2022.
8. Ian Rees, Ed Langley, Wah Chiu, and Steven J Ludtke. Emen2: an object oriented database and electronic lab notebook. *Microscopy and Microanalysis*, 19(1):1–10, 2013.
9. Thomas Flohr, M Prokop, C Becker, U Schoepf, A Kopp, R White, S Schaller, and B Ohnesorge. A retrospectively ecg-gated multislice spiral ct scan and reconstruction technique with suppression of heart pulsation artifacts for cardio-thoracic imaging with extended volume coverage. *European radiology*, 12:1497–1503, 2002.
