# Supplementary material for: TiltRec: an ultra-fast and open-source toolkit for cryo-electron tomographic reconstruction
Source: Bioinformatics. 2025 Feb 14;41(3):btaf068. doi: 10.1093/bioinformatics/btaf068 (PMC11886794; doi:10.1093/bioinformatics/btaf068)
Supplement: btaf068_Supplementary_Data [file btaf068_supplementary_data.zip › TiltRec_manual.pdf]

TiltRec: An open-source tool that utilizes GPU acceleration for efficient tomographic reconstruction

Contents

1 Overview **2**

2 Installation **2**

2.1 Prerequisites . . . 2

2.2 Configure the environment using Docker(Recommended). . . . 3

2.3 Executable file description . . . 3

3 Explanation of parameters and examples **4**

3.1 Parameter explanation of TiltRec . . . 4

3.2 Hdf to Mrc . . . 5

3.3 Explanation of output file . . . 5

3.4 Examples . . . 6

4 Integration with Upstream Alignment Tools **6**

4.1 IMOD . . . 7

4.2 RELION . . . 7

4.3 MarkAuto 2.0 . . . 7

5 Guidelines for Developers **8**

5.1 Unified memory . . . 8

5.2 Explanation of Key Variables and Functions in the ADMM Algorithm . . . 10

1 Overview

Here we present TiltRec, a GPU-accelerated tool for cryo-electron tomography reconstruction, designed for rapid and accurate three-dimensional reconstruction. TiltRec exhibits significant speed improvements compared to most current reconstruction software.

We provide users with four modes: y-axis non-accelerating, z-axis non-accelerating, y-axis slice-based accelerating, and z-axis unified memory accelerating. The y-axis and z-axis non-accelerating reconstruction mode supports three reconstruction methods: BPT, SIRT and SART. The y-axis slice-based accelerating reconstruction mode supports five methods: BPT, WBP, FBP, SIRT, and SART. The z-axis unified memory accelerating reconstruction mode supports six methods: BPT, WBP, FBP, SIRT, SART, and ADMM. TiltRec addresses the computational challenges in cryo-ET reconstruction, leveraging GPU acceleration to offer significant speed improvements. Additionally, it is fully open-source.

This manual introduces the instructions for installation (see section 2) and the detailed explanation of software parameters (see section 3).

2 Installation

The sections below explain how to download and install TiltRec on your computer.

We store the public release versions of TiltRec on GitHub, a site that provides code-development with version control and issue tracking through the use of git. To clone the repository, run the following command

```
git clone https://github.com/icthrm/TiltRec.git
```

2.1 Prerequisites

Note that TiltRec depends on and uses several external programs and libraries.

- Linux or Unix-like operating systems
- CMake
- OpenCV 4
- MPICH
- CUDA(Version above 11)

We have not identified any version dependencies for the above-mentioned software. For reference, below are the versions of our software: OpenCV 4.8.0, MPI 4.0, GCC 1.4, CMake 3.22.1, and CUDA 12.4.

To ensure compatibility and ease of setup, we highly recommend using Docker.

2.2 Configure the environment using Docker(Recommended).

For the convenience of environment configuration, we provide two compilation schemes: using Docker and not using Docker. Users who use Docker can directly install the external libraries described in Section 2.1 through Dockerfile, while users who do not use Docker must ensure that the server has correctly installed external libraries. The specific operations of both schemes are as follows:

- Using Docker

```
docker build -t tiltrec:v1 .
docker run -it --rm --gpus all tiltrec:v1 /bin/bash
mkdir build
cd build
cmake ..
make -j 8
```

- Not using Docker

```
mkdir build
cd build
cmake ..
make -j 8
```

2.3 Executable file description

After installation, four executable files will be generated, corresponding to four reconstruction modes, you can choose according to your needs:

- TiltRec-cuda: y-axis slice-based accelerating reconstruction

  Based on the y-axis acceleration reconstruction mode, it supports five methods: BPT, WBP, FBP, SIRT and SART. This mode divides the y-axis and uses GPU parallel processing, resulting in the fastest reconstruction speed and targeting practical users.

- TiltRec-mpi: y-axis non-accelerating reconstruction

Based solely on the MPI-accelerated mode, it supports three methods: BPT,WBP,FBP, SIRT and SART. This mode divides the y-axis based on CPU reconstruction, improving reconstruction speed and targeting practical users.

- TiltRecZ-cuda: z-axis unified memory accelerating reconstruction

Based on the z-axis unified memory accelerating reconstruction mode, it supports six methods: BPT, WBP, FBP, SIRT, SART and ADMM. This mode has a slower speed compared to y-axis slice-based accelerating reconstruction and is aimed at method developers.

- TiltRecZ-mpi: z-axis non-accelerating reconstruction

Based solely on the MPI-accelerated mode, it supports three methods: BPT,WBP,FBP, SIRT,SART and ADMM. This mode does not segment the 3D body, resulting in a slower reconstruction speed and is aimed at method developers.

3 Explanation of parameters and examples

3.1 Parameter explanation of TiltRec

==== Required options ====

- --input(-i)

The input tilt series path, where we assume that the tilt series has already been aligned and corrected for CTF. Supported formats include MRC and ST.

- --output(-o)

Designates the name of the resulting MRC or ST file.

- --tiltfile(-t)

The path of tilt angles, with supported formats including TLT and RAWTLT.

- --geometry(-g)

Defines geometric information of offset, pitch angle, z-axis offset, and thickness.

- --method(-m)

Selects the reconstruction method:

- Back Projection: BPT
- Filtered Back Projection: FBP
- Weighted Back Projection: WBP
- SART: SART, number of iterations, relaxation parameter
- SIRT: SIRT, number of iterations, relaxation parameter
- ADMM: ADMM, number of iterations, number of conjugate gradient iterations, relaxation parameter, threshold

==== Optional options ====

- --initial

Provides an initial MRC file to be used as the model for iterative reconstruction methods.

- --f2b

Output in byte format instead of float, to save memory usage, although this may result in a loss of precision.

- --help(-h)

Displays help information.

3.2 Hdf to Mrc

If the input and output are in HDF format, you can use the provided hdf2mrc and mrc2hdf scripts. Usage:

```
python script/hdf2mrc.py  Sar10453-10453_fin__bin4.hdf Sar10453-10453_fin__bin4.mrc
```

```
python script/mrc2hdf.py  Sar10453-10453_fin__bin4.mrc Sar10453-10453_fin__bin4.hdf
```

3.3 Explanation of output file

The output file is a 3D reconstruction result in MRC format, which can be opened and viewed in IMOD. The command is as follows:

```
imod outputfilename.mrc
```

3.4 Examples

When using TiltRec for 3D reconstruction, the necessary parameters provided by the user include the input MRC file, the resulting MRC file, the tilt angles file, the geometric information, and the reconstruction method. Other parameters are optional and users can choose according to their needs (see subsection 3.1). The following are four examples, corresponding to y-axis non-accelerating, y-axis slice-based accelerating, z-axis non-accelerating, and z-axis unified memory accelerating reconstruction.

- Example 1: y-axis non-accelerating reconstruction

```
mpirun -n 2 ./TiltRec-mpi --input ../../data/BBb/BBb_fin.mrc --output ../../data/BBb/BBb_output.mrc --tiltfile ../../data/BBb/BBb.rawtlt --geometry 0,0,0,300 --method BPT
```

- Example 2: y-axis slice-based accelerating reconstruction

```
mpirun -n 2 ./TiltRec-cuda --input ../../data/BBb/BBb_fin.mrc --output ../../data/BBb/BBb_output.mrc --tiltfile ../../data/BBb/BBb.rawtlt --geometry 0,0,0,300 --method WBP
```

- Example 3: z-axis non-accelerating reconstruction

```
mpirun -n 2 ./TiltRecZ-mpi --input ../../data/BBb/BBb_fin.mrc --output ../../data/BBb/BBb_output.mrc --tiltfile ../../data/BBb/BBb.rawtlt --geometry 0,0,0,300 --method SIRT,10,0.2
```

- Example 4: z-axis unified memory accelerating reconstruction

```
./TiltRecZ-cuda --input ../../data/BBb/BBb_fin.mrc --output ../../data/BBb/BBb_output.mrc --tiltfile ../../data/BBb/BBb.rawtlt --geometry 0,0,0,300 --method SART,10,0.2
```

4 Integration with Upstream Alignment Tools

To ensure a seamless workflow from raw tilt series to high-quality tomographic reconstructions, it is crucial to integrate TiltRec with widely used alignment tools. In this section, we illustrate how our software can be incorporated into existing pipelines that employ `IMOD`, `RELION`, and `MarkAuto 2.0` for tilt series alignment.

4.1 IMOD

In IMOD 4.11, Etomo's default format is the MRC file with the .mrc extension, though it also supports HDF files with the .hdf extension. Thus, after alignment, the files named *_ali.mrc or *_ali.hdf can be directly specified as the input via the --input option in TiltRec. The newly generated alignment angles, saved as a .tlt file, should be provided to TiltRec through the --tiltfile option.

4.2 RELION

We use RELION 5.0[1], where tilt series alignment is handled by the AlignTiltSeries workflow. Upon successful completion of this workflow, users can locate the alignment outputs in the corresponding job folder. The output logs typically include lines such as:

```
OutputTiltFile TS_01.tlt
OutputTransformFile TS_01.tltxf
```

The TS_01.tltxf file contains the linear transformations computed from the optimized camera parameters, while the TS_01.tlt file specifies the tilt angles associated with each projection. To generate the aligned tilt series, one can use IMOD with the newstack command:

```
newstack -input TS_01.mrc -output TS_01_ali.mrc -xf TS_01.tltxf
```

This approach applies the transformations to TS_01.mrc, resulting in the aligned file TS_01_ali.mrc. At this point, both TS_01_ali.mrc and the corresponding tilt angle file (TS_01.tlt) can be directly passed to TiltRec via the --input and --tiltfile options, respectively. This setup facilitates a streamlined workflow, enabling users to easily transition from RELION's alignment step to the reconstruction phase in TiltRec.

4.3 MarkAuto 2.0

MarkAuto[2] is another widely used tool for tilt series alignment, which similarly generates a transformation file (e.g., .xf) and a tilt-angle file (e.g., .tlt). After the alignment process, users can employ IMOD to apply the transformations and generate an aligned MRC file. For instance:

```
newstack -input TS_01.mrc -output TS_01_ali.mrc -xf TS_01.xf
```

This command creates the aligned tilt series TS_01_ali.mrc, which, together with the TS_01.tlt file, can be passed directly to TiltRec using the --input and --tiltfile options. This workflow ensures compatibility between MarkAuto and TiltRec, offering users a straightforward, end-to-end solution for tilt series alignment and subsequent reconstruction.

[1] https://relion.readthedocs.io/en/release-5.0/SPA_tutorial/InitialModel.html

[2] https://github.com/icthrm/Markerauto2.0

5 Guidelines for Developers

TiltRecZ-cuda and TiltRecZ-mpi are primarily designed for reconstruction methods requiring immediate access to the entire 3D volume, such as the ADMM algorithm or other iterative techniques that perform multiple passes over the full tomogram. To facilitate development efforts, this section introduces the unified memory technology employed in our approach and provides a detailed overview of the ADMM code structure.

5.1 Unified memory

Unified Memory, introduced by NVIDIA's CUDA architecture, is a memory management paradigm designed to streamline data sharing between the CPU (host) and GPU (device). Traditionally, separate memory spaces for host and device required explicit data transfers, introducing complexity and overhead in heterogeneous computing workflows. Unified Memory offers a single, shared memory address space, enabling both CPU and GPU to access data seamlessly. Through on-demand page migration and automatic memory coherence, the system dynamically transfers memory pages between host and device, ensuring efficient utilization of hardware resources. This abstraction significantly reduces the need for manual memory management, allowing developers to focus on algorithm design rather than low-level memory operations.

In cryo-ET tomographic reconstruction workflows, certain reconstruction algorithms require access to the entire three-dimensional (3D) volume simultaneously to perform global optimizations or iterative refinements. However, GPU memory limitations often prevent large 3D volumes from being fully loaded onto the device. Unified Memory provides an effective solution by enabling dynamic memory page migration between the CPU and GPU, allowing the reconstruction algorithm to transparently access the required data without being constrained by GPU memory capacity. This not only simplifies memory management but also facilitates the development of more advanced reconstruction techniques that would otherwise be limited by hardware constraints.

Next, we will present an example from NVIDIA's official documentation, followed by a simple demonstration showcasing how Unified Memory is utilized in our TiltRec implementation. This aims to provide better reference and guidance for other developers.

Official Resources

For more information, refer to the official NVIDIA resources:

- **CUDA C++ Programming Guide**: A comprehensive guide detailing Unified Memory concepts, mechanisms, and best practices. Available at: docs.nvidia.com

- **Introduction to CUDA Unified Memory**: An in-depth technical blog explaining Unified Memory, its benefits, and application scenarios. Available at: developer.nvidia.com

Unified Memory in TiltRec

In our TiltRecZ-cuda implementation, Unified Memory is particularly beneficial for large-scale tomographic reconstructions where the entire 3D volume cannot fit entirely into GPU memory. Below is a simplified demo showing how we leverage Unified Memory:

1. Unified Memory Allocation Unified memory is allocated using `cudaMallocManaged` for `vol.data` and `originalProjsData`. This allows the GPU and CPU to share the same memory region, avoiding explicit data copying.

```
cudaMallocManaged((void **)(&vol.data), sizeof(float) * vol.X() * vol.Y() * vol.Z());
cudaMallocManaged((void **)(&originalProjsData), sizeof(float) * projs.X() * projs.Y() * projs.Z());
```

2. Data Transfer to Device The projection data and parameters are transferred from the host to the device using `cudaMemcpy`.

```
cudaMemcpy(cudev.coeffs, &(params[0]), sizeof(SimCoeff) * params.size(), cudaMemcpyHostToDevice);
cudaMemcpy(cudev.origin, &origin, sizeof(Point3DF), cudaMemcpyHostToDevice);
```

3. Kernel Execution The `CuBackProjKernelZ` kernel is executed on the GPU for backprojection operations. After the kernel completes, the data in `vol.data` is updated and can be directly written back to disk.

```
CuBackProjKernelZ<<<dim3Grid, dimBlock>>>(cudev.origin, cudev.coeffs,
                                          vol.data, originalProjsData, cudev.x,
                                          cudev.y, z);
```

4. Data Write-back Since unified memory allows the CPU and GPU to share the same memory, the write-back operation can directly use the shared memory.

```
mrcvol.WriteBlock<float>(0, vol.Z(), 'z', vol.data);
```

5. Memory Cleanup Finally, the allocated unified memory is freed to avoid memory leaks.

```
cudaFree(vol.data);
cudaFree(originalProjsData);
```

5.2 Explanation of Key Variables and Functions in the ADMM Algorithm

1. Main Variables

In our program, the derived expressions are as follows, where the target 3D volume to be reconstructed is denoted as **V**. The following are key variables in the algorithm:

- `struct CuTaskDataZ` // **Task Data Structure containing all necessary parameters**
  - `Point3DF *origin;` // **Original coordinates of the voxels**
  - `SimCoeff *coeffs;` // **Projection coefficients array**
  - `float *slice;` // **Storage for slice data, for intermediate use**
  - `float *projs;` // **Storage for image data, for intermediate use**
  - `float *s;` // **Intermediate variable used in computations**
  - `float *c;` // **Intermediate variable used in computations**
  - `int nproj;` // **Number of projections (images)**
  - `int x;` // **X-dimension size of the volume to be reconstructed**
  - `int y;` // **Y-dimension size of the volume to be reconstructed**
  - `int z;` // **Thickness of the volume to be reconstructed**
- `*htb:` // **Stores the computed results of H**
- `*uk:` // **Optimization parameter in the ADMM algorithm for** u_k
- `*dk:` // **Optimization parameter in the ADMM algorithm for** d_k
- `*x0:` // **Intermediate variable used to store the result of the computation of** x_0 **in ADMM**

2. Key Functions

2.1 CuAtb_ADMM_Z

Purpose: This function performs a computational step in the ADMM algorithm responsible for calculating **H**. Specifically, it computes the backprojection results in parallel on the GPU.

Input Parameters:

- `*origin:` Original coordinate positions used for coordinate transformation.
- `*coeffs:` Coefficient array used for projection calculations.

- `*htb`: Array used to store the backprojection results.
- `cudev_x`: X-dimension size of the volume.
- `cudev_y`: Y-dimension size of the volume.
- `*projsdata`: Image data.
- `coordz_offset`: Direction offset, default is 0.
- `angIdxStart`: Projection angle index.

Output Parameters: This function does not return a value directly but updates the contents of the `*htb` array through computation.

2.2 CuATbGammaIt_ADMM_Z

Purpose: This function performs a specific step in the ADMM algorithm by calculating the result of **H** and updating `*htb` based on the gamma parameter.

Input Parameters:

- `*htb`: The previously computed `*htb` array.
- `*u_k`: Variable in the ADMM iteration.
- `*d_k`: Variable in the ADMM iteration.
- `volsize`: Size of the 3D volume.
- `gamma`: A constant parameter used to adjust the strength of the update.

Output Parameters: The function updates the `*htb` array.

2.3 CuATaGammaI_ADMM_Z

Purpose: This function executes another step in the ADMM iteration, specifically calculating **H** and updating intermediate results.

Input Parameters:

- `*origin`: Original coordinate positions used for coordinate transformation.
- `*coeffs`: Coefficient array used for projection calculations.
- `*a_x`: Intermediate variable to store computation data.
- `*w`: Intermediate variable to store computation data.

- `*x0`: Used to store the computed result of x_0 in ADMM.
- `cudev_x`: X-dimension size of the volume.
- `cudev_y`: Y-dimension size of the volume.
- `volsize`: Size of the volume.
- `*voldata`: Current volume data.
- `gamma`: Adjustment parameter.
- `dim_1grid, dim_3grid, dim_block`: GPU parallel parameters for grid and block size during parallel computations.
- `coordz_offset`: Direction offset, default is 0.
- `projsnum`: Number of images.

Output Parameters: The function updates the `*x0` array.

2.4 CuApplycg__ADMM__Z

Purpose: This function implements the Conjugate Gradient (CG) method for solving linear systems and optimizes volume data during the current ADMM iteration.

Input Parameters:

- `&cudevice`: Task data structure containing all necessary parameters.
- `*voldata`: Current volume data.
- `*x0`: Previously computed result of x_0.
- `*htb`: Previously computed result of **H**.
- `numberIteration`: Number of iterations in the Conjugate Gradient method.
- `gamma`: Adjustment parameter.
- `volsize`: Size of the 3D volume.
- `dim_1grid, dim_3grid, dim_block`: GPU parallel parameters for grid and block size during parallel computations.
- `projsnum`: Number of images.

Output Parameters: The function updates the volume data (`*voldata`).

2.5 CuSoft_ADMM_Z

Purpose: This function performs the soft thresholding operation in ADMM to update $\mathbf{u}_k$.

Input Parameters:

- `*u_k:` Variable to be optimized in the ADMM iteration.
- `*d_k:` Variable in the ADMM iteration.
- `*voldata:` Current volume data.
- `soft:` A constant soft threshold value.
- `volsize:` Size of the 3D volume.

Output Parameters: The function updates the `*u_k` array.

2.6 Cu_dk_ADMM_Z

Purpose: This function performs the update operation for the variable $\mathbf{d}_k$.

Input Parameters:

- `*d_k:` Variable d_k to be updated.
- `*u_k:` Previously updated u_k.
- `*voldata:` Current volume data.
- `volsize:` Size of the 3D volume.

Output Parameters: The function updates the `*d_k` array.

3. Developer Guidelines

Based on the derived expressions, the following sections outline how to call or modify the Conjugate Gradient (CG) or ADMM methods in the program.

3.1 Conjugate Gradient (CG)

The Conjugate Gradient (CG) method is an optimization technique used to solve linear systems. In this implementation, the function `CuApplycg_ADMM_Z` is used to optimize the volume data via CG in each ADMM iteration. Developers wishing to use this method for similar problems should first compute x_0 (the corresponding result in this program) and $\mathbf{H}$ (the corresponding result in this program), and then pass x_0 and $\mathbf{H}$ to the `CuApplycg_ADMM_Z` function.

Additionally, the number of iterations in CG can be controlled via the `numberIteration` variable. By default, `CuApplycg_ADMM_Z` implements a fixed number of iterations. To improve the convergence of CG, developers can adjust the step size or stopping criteria. For instance, introducing dynamic stopping conditions (such as a convergence threshold) can be done by modifying the code logic as needed.

3.2 ADMM Method

The ADMM (Alternating Direction Method of Multipliers) method decomposes a complex optimization problem into smaller subproblems, which are solved iteratively. In this program, the parameters to be optimized are specified, and developers should clearly identify the parameters that need to be optimized in their specific problem and correctly pass these parameters to the program.

Each key computational step in the algorithm has an associated update operation. If improvements to the ADMM process are required, the specific computations in each step can be adjusted according to the actual problem at hand. Additionally, the `iteration` variable can be adjusted to control the total number of iterations in the ADMM process. Developers can also select different threshold values for `soft`, adjust the `gamma` parameter, or, if necessary, modify the order of updates between different steps.

Table 1: Runtime Comparison for Tomographic Reconstruction Algorithms within Different Tools (seconds).

2*Method	2*Software	BBb				
1024 × 1024 × 300	EMPAIR-10453					
5760 × 4092 × 300	EMPIAR-10045					
3838 × 3710 × 1081	EMPIAR-10164					
7420 × 7676 × 300	2* Speedup3					
SIRT(30)	TiltRec-cuda	**18.126**	**395.777**	**860.360**	**987.638**	-
	TiltRecZ-cuda	46.563	24655.766	72555.030	826936.478	246.6
	IMOD	55.515	962.735	1848.176	1471.093	2.3
	TOMO3D	237.412	4126.462	6030.4192	2132.804	8.1
FBP	TiltRec-cuda	2.844	282.489	801.271	901.382	-
	TiltRecZ-cuda	**2.843**	730.725	1750.031	15923.281	5.9
	IMOD	4.178	**254.797**	**714.634**	**852.521**	1.1
	TOMO3D	9.307	452.6952	1018.2589	1002.953	1.8
WBP	TiltRec-cuda	**3.567**	275.172	**820.074**	932.382	-
	TiltRecZ-cuda	3.565	712.965	1505.381	13029.407	4.8
	IMOD	4.367	**250.408**	837.113	**890.580**	1.0
	TOMO3D	6.018	458.216	1070.4727	932.953	1.4
SART(10)	TiltRec-cuda	**9.816**	**272.983**	**833.109**	**946.145**	-
	TiltRecZ-cuda	16.442	5586.373	17851.595	241372.138	74.7
	TiltRec-mpi	15054.243	351940.342	291384.293	94179.393	818.0
	TiltRecZ-mpi	9828.245	301141.736	284105.285	88103.291	634.6
BPT	TiltRec-cuda	2.984	**248.065**	**735.503**	**822.379**	-
	TiltRecZ-cuda	**2.001**	785.921	1047.275	12854.158	5.2
	TiltRec-mpi	864.316	27360.118	26008.667	59166.720	126.8
	TiltRecZ-mpi	819.819	26574.536	26013.294	59166.720	122.3
ADMM(10)	TiltRecZ-cuda	**121.370**	**11067.784**	**789.389**1	**1200.171**	-
	TiltRecZ-mpi	30385.100	646805.701	44118.0001	176041.0282	-
	Relion	-	-	-	1726.4292	

1 indicates that result obtained from running on a dataset down-sampled by a factor of two.

2 indicates that result obtained from running on a dataset down-sampled by a factor of four.

3 the mean ratio of the runtime of other software to that of TiltRec-cuda across each dataset.
